# Design, Fabrication and Experiment of Double U-Beam MEMS Vibration Ring Gyroscope

**DOI:** 10.3390/mi10030186

**Published:** 2019-03-13

**Authors:** Huiliang Cao, Yu Liu, Zhiwei Kou, Yingjie Zhang, Xingling Shao, Jinyang Gao, Kun Huang, Yunbo Shi, Jun Tang, Chong Shen, Jun Liu

**Affiliations:** 1 Key Laboratory of Instrumentation Science & Dynamic Measurement, Ministry of Education, North University of China, Tai Yuan 030051, China; caohuiliang@nuc.edu.cn (H.C.); nucliuyu@126.com (Y.L.); kouzhiwei@imut.edu.cn (Z.K.); 1302034243@st.nuc.edu.cn (Y.Z.); huanying3557913@sina.com (X.S.); gjy.1001@163.com (J.G.); ffthk@nuc.edu.cn (K.H.); shiyunbo@nuc.edu.cn (Y.S.); tangjun16@126.com (J.T.); 2 Science and Technology on Electronic Test & Measurement Laboratory, North University of China, Tai Yuan 030051, China

**Keywords:** MEMS gyroscope, double-U ring structure, structure design, FEA simulation, fabrication, experiment

## Abstract

This study presents a new microelectromechanical system, a vibration ring gyroscope with a double U-beam (DUVRG), which was designed using a combination of mathematical analysis and the finite element method. First, a ring vibration resonator with eight double U-beam structures was developed, and 24 capacitive electrodes were designed for drive and sense according to the advantageous characteristics of a thin-shell vibrating gyroscope. Then, based on the elastic mechanics and thin-shell theory, a mathematical stiffness model of the double U-beam was established. The maximum mode resonant frequency error calculated by the DUVRG stiffness model, finite element analysis (FEA) and experiments was 0.04%. DUVRG structures were manufactured by an efficient fabrication process using silicon-on-glass (SOG) and deep reactive ion etching (DRIE), and the FEA value and theoretical calculation had differences of 5.33% and 5.36% with the measured resonant frequency value, respectively. Finally, the static and dynamic performance of the fabricated DUVRG was tested, and the bias instability and angular random walk were less than 8.86 (°)/h and 0.776 (°)/√h, respectively.

## 1. Introduction

Due to the advantages on small volume, low power consumption and easy integration, MEMS (microelectromechanical system) gyroscopes, devices and technologies are utilized in more and more civil and military application areas, including the fields of aircraft and vehicle control, automotive safety, energy harvesting, industrial controlling, inertial navigation, attitude determination, micro robot, micro signal detection, equipment fault diagnosis and consumer electronics [1,2,3,4,5,6,7,8,9,10,11]. A lot of work has been reported to improve the gyro characteristic, including bias drift prediction [12], tiny capacitance detection interface circuit [13], structure noising analysis [14], quality factor optimization [15], structure advanced manufacture [16], bandwidth expanding [17,18], data compensation [19,20,21], quadrature error correction [22] and so on.

MEMS vibratory gyroscopes can be divided into capacitance detection, current detection, resistance detection and piezoelectric sense, according to different sense methods [4]. Among gyroscopes composed of sensitive structures, only the axisymmetric structure can achieve high precision. The axisymmetric structure includes the cylinder-shaped and bell-shaped [23,24,25,26,27]. Among them, the consumer MEMS gyro mostly adopts the traditional tuning fork structure, which cannot meet the application requirements of high precision and special environments. The military high-end MEMS gyro technology is completely different from the commercial MEMS gyro technology. Military high-end MEMS gyro technology generally adopts a full-symmetric structure because it has good environmental adaptability. For example, the US DARPA micro-PNT project focuses on the development of multi-ring disk resonator gyroscopes (DRGs) and micro hemisphere resonator gyroscopes (HRGs). At present, existing high-precision gyroscopes are mostly hemispherical resonant gyroscopes (HRGs). Compared with current MEMS gyro technology such as the tuning tuning fork type, flat vibration type and shell vibration type, HRGs have the advantages of high precision, high dynamic range, strong anti-overload resistance, can directly measure the rotation angle and is convenient for mass production. It becomes an effective technology for many kinds of medium and high precision weapon carrier platforms in the future, especially for inertial navigation systems of high dynamic rotating guidance projectiles and rockets. However, HRGs are difficult to mass produce due to complex processing because of their three-dimensional structure [28]. Due to the difficulty in manufacturing hemispherical resonator gyros and high assembly requirements, it is difficult to achieve mass production. Both the vibrating ring gyroscopes (VRGs) and the HRGs work based on the inertial effect of the elastic wave, and the specific vibration form is the circular-elliptical bending vibration of the resonant. The VRGs are the two-dimensional representation of the HRGs that can reduce the difficulty of the manufacturing while ensuring the same vibration form as the HRGs. The vibrating ring gyroscopes (VRGs), which are very suitable for current MEMS manufacturing processes, is a simplified form of the HRG, and VRG can be mass produced at low cost. The main component of the VRGs are the ring-shaped resonator without rotational vibration. Because of the advantages of the materials and structures of ring resonators, VRGs have many significant advantages such as high precision, low energy consumption, long life and mass production. Therefore, based on MEMS technology, the research on the technical innovation of VRGs and the development of new MEMS solid wave gyro with high precision and good impact resistance is of great significance for promoting the rapid development of strategic weapon equipment and high-end navigation systems.

The VRG is an ideal choice for a high-performance gyroscope. To date, the VRG has garnered the attention of many researchers. For example, Ayazi proposed a kind of fully symmetrical ring gyroscope with a high aspect ratio structure, with a quality factor of 1200, drive mode amplitude of 0.15 µm and resolution of 1 (°)/s [29,30]. Yoon Sungjin investigated a MEMS VRG with high shock reliability, and the bias instability, scale factor accuracy and angular random walk (ARW) were 0.76 (°)/h, 27 ppm and 0.078 (°)/√s, respectively [31]. Liu Jili proposed a novel ring vibration gyroscope with electromagnetic electrodes based on SOI [32]. Zaman performed a resonance star structure improvement, and the gyroscope bias stability reached 2.5 (°)/h [33]. Hu Zhongxu presented a parametrically amplified MEMS ring gyroscope with an improved scaling factor and signal to noise ratio (SNR) by way of parametric amplification [34]. Tao Yi proposed a kind of metal ring gyroscope with piezoelectric electrodes, its quality factor approximately 5000 in atmosphere and a bias instability of 1.5 (°)/h [35]. Zhou Xin reported a kind of metal ring gyroscope with piezoelectric electrodes whose quality factor is approximately 5000 in atmosphere and the zero-bias instability about 1.5 (°)/h at room temperature [36]. A novel S-spring ring MEMS gyroscope was proposed by Kou Zhiwei, and the structure was fabricated by Silicon-on-Glass (SOG) technology [37]; the bias stability and angle random walk are 0.0119 (°)/s and 0.0359 (°)/√s [38].

In this paper, we designed a novel vibrating ring gyroscope with a double U-beam (DUVRG), and this work included the design, modeling, manufacturing and experiments. This paper continues as follows: In Section 2, the structural characteristics and working principle of a DUVRG are introduced. In Section 3, the stiffness model of the U-beam is established based on the elastic mechanics and thin-shell theory, and the DUVRG model is established in finite element analysis (FEA) software, and accurate analysis results are obtained. Section 4 describes the fabrication process used for prototypes. In Section 5, a prototypal gyroscope is selected for preliminary experiments. Finally, some important conclusions and discussions are drawn in Section 6.

## 2. MEMS DUVRG Working Principle

### 2.1. MEMS DUVRG Structure

**Figure 1:**
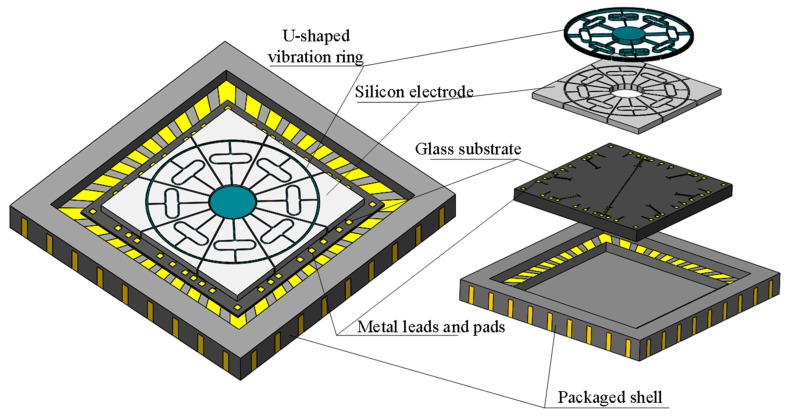
Double U-beam (DUVRG) structure chip diagram.

A schematic of the presented symmetric capacitive MEMS VRG with a double U-beam (DUVRG) is shown in Figure 1. The DUVRG is composed of a fully symmetric ring resonator supported by a central anchor point, a glass substrate with patterned electrode leads and twenty-four silicon capacitor electrodes with control and tuning. Eight double U-shaped symmetrical supporting springs connect the outer ring resonator and the center anchor point.

**Figure 2:**
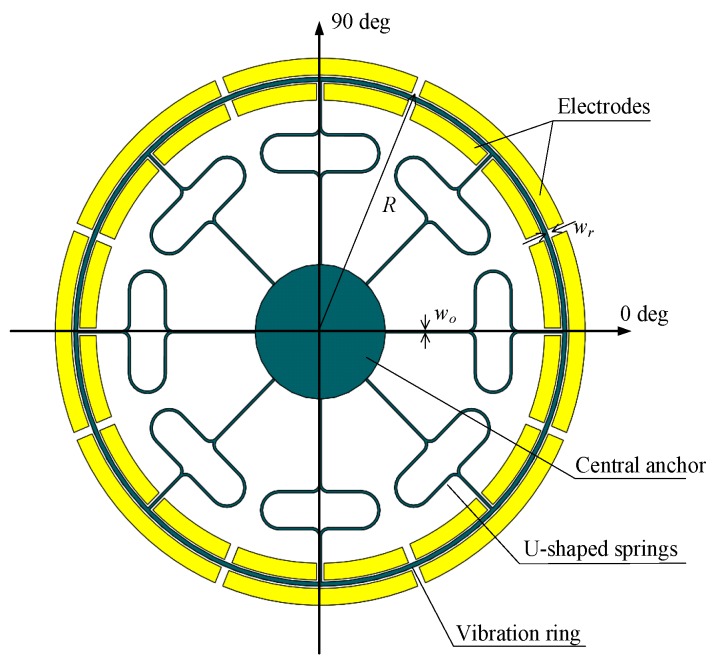
DUVRG resonant structure diagram.

The patterned electrode leads are connected to the peripheral interface circuitry. Referring to the distribution of the drive and sense electrodes of hemispherical resonant gyroscopes (HRGs), the external electrodes of the DUVRG can be evenly distributed around the ring, and the distribution diagram is shown in Figure 2.

### 2.2. MEMS DUVRG Working Principle

**Figure 3:**
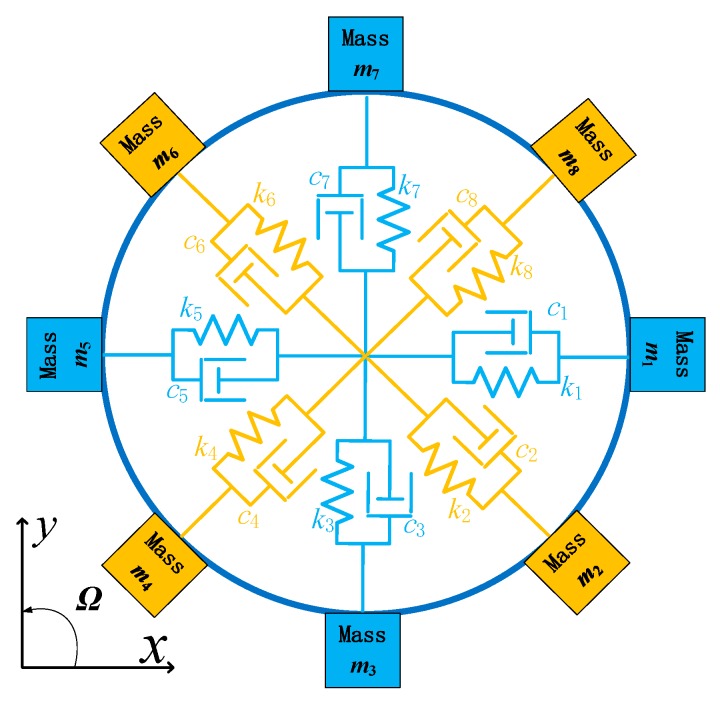
DUVRG resonant structure mechanical equivalent model.

The DUVRG has two working modes: the drive mode and the sense mode. Both of these modes can be regarded as mass-spring-damper second-order vibration systems. A basic mechanical equivalent model is shown in Figure 3.

**Figure 4:**
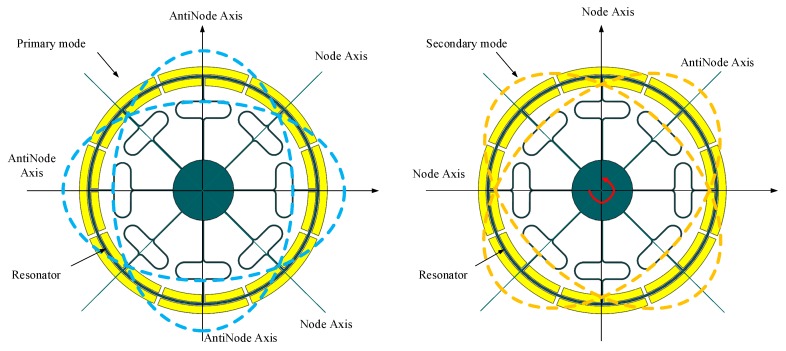
DUVRG resonant structure drive mode (left) and sense mode (right).

The drive mode (primary mode) resonant form is shown in Figure 4 (left picture). Under the normal working conditions of the DUVRG, the ring resonator is forced to vibrate at the drive mode resonant frequency of the structure along the blue imaginary line under the action of a periodic drive force. The movement of the structure is the structure shape change, which is a neat symmetry structure by antinode axis. When an angular rate Ω (red line in Figure 4) is input in the *Z*-axis direction perpendicular to the direction of the forced vibration, the ring resonator vibrates following the orange imaginary line (in Figure 4 right picture) direction in the third direction perpendicular to the directions of the forced vibration and angular velocity input.

According to the principle of vibration mechanics, the dynamic equation of the ring resonator is given by

(1) $$\begin{array}{l} M_{x}\ddot{x}+C_{x}\dot{x}+K_{x}x=F_{x}\\ M_{y}\ddot{y}+C_{y}\dot{y}+K_{y}y=-F_{c} \end{array}$$

where ***M_x_*** and ***M_y_***, ***C_x_*** and ***C_y_***, ***K_x_*** and ***K_y_***, ***F_x_*** and ***F_C_***, ***x*** and ***y*** are matrixes of the equivalent masses, equivalent damping coefficients, equivalent stiffness coefficients, drive and Coriolis force, displacement of drive and sense modes, respectively. And their matrixes can be written as

(2) $$\left\{ \begin{array}{l} M_{x}=\mathrm{diag}\left[\begin{array}{cccccccc} m_{1} & 0 & m_{3} & 0 & m_{5} & 0 & m_{7} & 0 \end{array}\right]\\ M_{y}=\mathrm{diag}\left[\begin{array}{cccccccc} 0 & m_{2} & 0 & m_{4} & 0 & m_{6} & 0 & m_{8} \end{array}\right]\\ C_{x}=\mathrm{diag}\left[\begin{array}{cccccccc} c_{1} & 0 & c_{3} & 0 & c_{5} & 0 & c_{7} & 0 \end{array}\right]\\ C_{y}=\mathrm{diag}\left[\begin{array}{cccccccc} 0 & c_{2} & 0 & c_{4} & 0 & c_{6} & 0 & c_{8} \end{array}\right]\\ K_{x}=\mathrm{diag}\left[\begin{array}{cccccccc} k_{1} & 0 & k_{3} & 0 & k_{5} & 0 & k_{7} & 0 \end{array}\right]\\ K_{y}=\mathrm{diag}\left[\begin{array}{cccccccc} 0 & k_{2} & 0 & k_{4} & 0 & k_{6} & 0 & k_{8} \end{array}\right]\\ F_{x}=\mathrm{diag}\left[\begin{array}{cccccccc} f_{1} & 0 & f_{3} & 0 & f_{5} & 0 & f_{7} & 0 \end{array}\right]\\ F_{C}=\mathrm{diag}\left[\begin{array}{cccccccc} 0 & f_{C2} & 0 & f_{C4} & 0 & f_{C6} & 0 & f_{C8} \end{array}\right] \end{array}\right.$$

where *m_i_*, *c_i_*, *k_i_*, *f_i_* and *f_Ci_* are the equivalent mass, the damping coefficient, the stiffness coefficient, the drive force and the Coriolis force in the direction of the eight support beams of the DUVRG.

The parameters for each direction of the four drive support beams and the four sense support beams of the DUVRG are the same in size and different in direction. Therefore, in the analysis, it is possible to analyze only one pair of systems. For this analysis, *m*_1_ is selected in the drive direction, and *m*_2_ is selected in the sense direction. Thus, the second-order dynamic equation of the gyroscope is as follows:

(3) $$\begin{array}{l} m_{1}\frac{d^{2}}{dt^{2}}x+c_{1}\frac{d}{dt}x+k_{1}x=f_{1}\\ m_{2}\frac{d^{2}}{dt^{2}}y+c_{2}\frac{d}{dt}y+k_{2}y=-2m_{1}\Omega \dot{x} \end{array}$$

The drive force is set as follows:

(4) $$f_{1}=A_{F}\sin\omega_{d}t$$

where *A_F_* is the amplitude of the drive force and *ω_d_* is the drive frequency of the electrostatic force. Using Equations (1) and (2), the equation of motion for the drive mode can be expressed as

(5) $$m_{1}\ddot{x}+c_{1}\dot{x}+k_{1}x=A_{F}\sin\omega_{d}t$$

In addition, the natural frequency and the damping ratio of the drive mode can be determined as follows:

(6) $$\omega_{n1}=\sqrt{\frac{k_{1}}{m_{1}}},\;\xi_{1}=\frac{c_{1}}{2m_{1}\omega_{n1}}$$

By solving the differential equation, the displacement of the vibration mass in the drive direction can be obtained as

(7) $$\begin{array}{ll} x(t) & =\frac{2A_{F}\xi_{1}\omega_{n1}\omega_{d}/m_{1}}{{\left(\omega_{n1}^{2}-\omega_{d}^{2}\right)}^{2}+4\xi_{1}^{2}\omega_{n1}^{2}\omega_{d}^{2}}e^{-\xi_{1}\omega_{n1}t}\cos(\omega_{n1}t\sqrt{1-\xi_{1}^{2}})\\ & +\frac{A_{F}\omega_{d}(2\xi_{1}^{2}\omega_{n1}^{2}+\omega_{d}^{2}-\omega_{n1}^{2})/m_{1}}{\omega_{n1}\sqrt{1-\xi_{1}^{2}}[{\left(\omega_{n1}^{2}-\omega_{d}^{2}\right)}^{2}+4\xi_{1}^{2}\omega_{n1}^{2}\omega_{d}^{2}]}e^{-\xi_{1}\omega_{n1}t}\sin(\omega_{n1}t\sqrt{1-\xi_{1}^{2}})\\ & +\frac{A_{F}/m_{1}}{\omega_{n1}^{2}\sqrt{{\left(1-\frac{\omega_{d}^{2}}{\omega_{n1}^{2}}\right)}^{2}+{\left(2\xi_{1}\frac{\omega_{d}}{\omega_{n1}}\right)}^{2}}}\sin(\omega_{d}t-\varphi_{1}) \end{array}$$

Since the motion of the vibration mass in the drive direction is the combined motion of the attenuation motion and the simple harmonic motion, the displacement of the mass of the gyroscope in the drive direction during stable operation can be determined as

(8) $$x(t)=A_{1}\sin(\omega_{d}t-\varphi_{1})$$

where the phase and amplitude can be expressed as:

$$\varphi_{1}=\arctan\frac{2\xi_{1}\omega_{n1}\omega_{d}}{\omega_{n1}^{2}-\omega_{d}^{2}},\;A_{1}=\frac{A_{F}/m_{1}}{\omega_{n1}^{2}\sqrt{{\left(1-\frac{\omega_{d}^{2}}{\omega_{n1}^{2}}\right)}^{2}+{\left(2\xi_{1}\frac{\omega_{d}}{\omega_{n1}}\right)}^{2}}}$$

when the gyroscope input axis is rotated at an angular velocity Ω relative to the inertia space, the Coriolis force generated in the sense direction is given by the following:

(9) $$F_{C}=2m_{2}\Omega \times \dot{x}=2m_{2}\Omega A_{1}\omega_{d}\cos(\omega_{d}t-\varphi_{1})$$

In open-loop detection, the equation of motion of the vibration mass in the sense direction is written as

(10) $$F_{C}=m_{2}y+c_{2}y+k_{2}y=2m_{2}\Omega A_{1}\omega_{d}\cos(\omega_{d}t-\varphi_{1})=B_{F}\cos(\omega_{d}t-\varphi_{1})$$

where, *B_F_* = 2*m*_2_Ω*A*_1_*ω_d_*, the natural frequency and the damping ratio of the sense mode can be determined as follows:

(11) $$\omega_{n2}=\sqrt{\frac{k_{2}}{m_{2}}},\;\xi_{2}=\frac{c_{2}}{2m_{2}\omega_{n2}}$$

By solving the differential equation, the displacement of the vibration mass in the sense direction can be obtained as follows:

(12) $$\begin{array}{l} y(t)=\frac{B_{F}[2\xi_{2}\omega_{n2}\omega_{d}\sin\varphi_{1}+(\omega_{n2}^{2}-\omega_{d}^{2})\cos\varphi_{1}]/m_{2}}{{\left(\omega_{n2}^{2}-\omega_{d}^{2}\right)}^{2}+4\xi_{2}^{2}\omega_{n2}^{2}\omega_{d}^{2}}e^{-\xi_{2}\omega_{n2}t}\cos(\omega_{n2}t\sqrt{1-\xi_{2}^{2}})\\ -\frac{B_{F}[\xi_{2}\omega_{n2}(\omega_{n2}^{2}-3\omega_{d}^{2})\cos\varphi_{1}+\omega_{d}(2\xi_{2}^{2}\omega_{n2}^{2}+\omega_{n2}^{2}-\omega_{d}^{2})\sin\varphi_{1}]/m_{2}}{\omega_{n2}t\sqrt{1-\xi_{2}^{2}}[{\left(\omega_{n2}^{2}-\omega_{d}^{2}\right)}^{2}+4\xi_{2}^{2}\omega_{n2}^{2}\omega_{d}^{2}]}e^{-\xi_{2}\omega_{n2}t}\sin(\omega_{n2}t\sqrt{1-\xi_{2}^{2}})\\ +\frac{B_{F}/m_{2}}{\omega_{n2}^{2}\sqrt{{\left(1-\frac{\omega_{d}^{2}}{\omega_{n2}^{2}}\right)}^{2}+{\left(2\xi_{2}\frac{\omega_{d}}{\omega_{n2}}\right)}^{2}}}\cos(\omega_{d}t-\varphi_{1}-\varphi_{2}) \end{array}$$

Since the movement of the vibration mass in the sense direction is also the combined motion of the attenuation motion and the simple harmonic motion, the displacement of the gyroscope in the sense direction can be obtained after applying the sensitivity of the mass to the Coriolis force as follows:

(13) $$y(t)=A_{2}\cos(\omega_{d}-\varphi_{1}-\varphi_{2})$$

In addition, the phase and the amplitude of the sense mode can be obtained as per

(14) $$\begin{array}{c} \varphi_{2}=\arctan\frac{2\xi_{2}\omega_{n2}\omega_{d}}{\omega_{n2}^{2}-\omega_{d}^{2}},\\ A_{2}=\frac{2A_{F}\omega_{d}\Omega }{m_{1}}\cdot \frac{1}{\omega_{n1}^{2}\sqrt{{\left(1-\frac{\omega_{d}^{2}}{\omega_{n1}^{2}}\right)}^{2}+{\left(2\xi_{1}\frac{\omega_{d}}{\omega_{n1}}\right)}^{2}}}\cdot \frac{1}{\omega_{n2}^{2}\sqrt{{\left(1-\frac{\omega_{d}^{2}}{\omega_{n2}^{2}}\right)}^{2}+{\left(2\xi_{1}\frac{\omega_{d}}{\omega_{n2}}\right)}^{2}}} \end{array}$$

## 3. MEMS DUVRG Structure Design

### 3.1. DUVRG Stiffness Model

**Figure 5:**
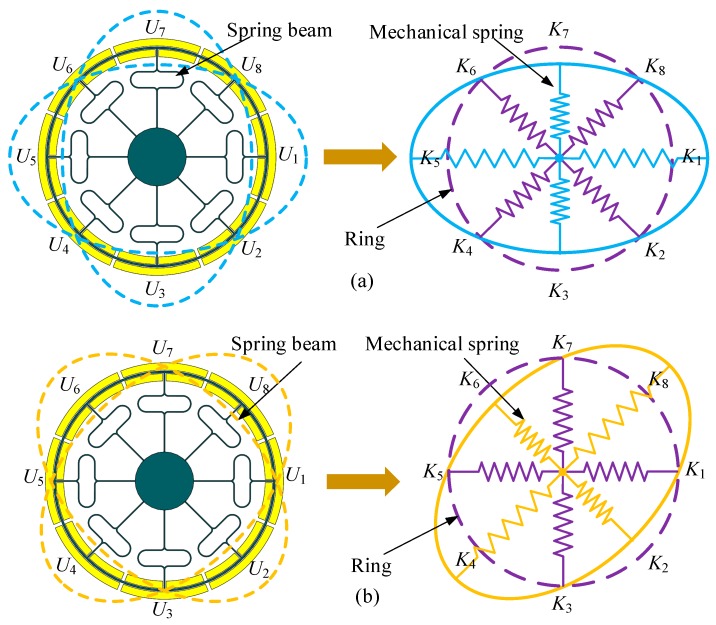
(**a**) DUVRG drive mode stiffness model. (**b**) DUVRG sense mode stiffness model.

The DUVRG is a centrally symmetrical thin-shell element, and the thin shell is mainly subjected to radial stretching and bending. Combined with the characteristics of the ring resonator structure, the wall thickness of the DUVRG is far less than its radius, so the vibration of the ring resonator can be analyzed by shell theory. Since the central anchor of the DUVRG is bonded to the glass substrate by electrostatic bonding, the axial movement of the structure is neglected in the DUVRG model. By analyzing the stiffness of the ring resonator in the *X-Y* plane, it is possible to assume eight springs at the nodes and antinodes of the ring resonator, as shown in Figure 5. When the ring resonator is in the drive mode, the springs *U*_1_, *U*_3_, *U*_5_ and *U*_7_ are stretched or compressed; when the ring resonator is in the sense mode, the springs *U*_2_, *U*_4_, *U*_6_ and *U*_8_ are stretched or compressed.

From Equation (1), the resonant frequencies of the system can be expressed as

(15) $$\omega_{1,2}=\sqrt{\frac{1}{2}\left(\frac{k_{11}}{m_{1}}+\frac{k_{22}}{m_{2}}\right)\pm \sqrt{{\left(\frac{k_{11}}{m_{1}}-\frac{k_{22}}{m_{2}}\right)}^{2}+\frac{4k_{12}}{m_{1}m_{2}}}}$$

where *k_ij_* (*i*, *j* = 1, 2) are the elements of the coupling mechanical stiffness between the drive and sense modes (usually *k*_12_ = *k*_21_). In an ideal VRG, *m*_1_ = *m*_2_ = *m*, *k*_11_ = *k*_22_ = *k*, and *k*_12_ = *k*_21_ = 0.

The working mode of the DUVRG is an in-plane bending vibration, and the double U-shaped elastic supporting beam is mainly stretched or compressed. According to material mechanics and elastic mechanics, the strain energy is mainly composed of the axial tensile strain potential energy and the potential energy of the bending strain. The strain energy and radial displacement of the elastic beam are

(16) $$\left\{ \begin{array}{l} U=\int \frac{P^{2}(x)}{2ES}+\int \frac{M^{2}(x)}{2EI_{o}}\\ \delta =\int \frac{P(x)}{ES}\cdot \frac{\partial P(x)}{\partial F}+\int \frac{M(x)}{EI_{o}}\cdot \frac{\partial M(x)}{\partial F}dx \end{array}\right.$$

where *E* is the elastic modulus of the silicon material, *S* is the cross-sectional area of the elastic beam, *P(x)* is the axial tensile force of the elastic beam, *M(x)* is the bending moment of the elastic beam and *I*_o_ is the moment of inertia.

In the 1–2 section of the elastic beam, the axial tensile force *P*_1_*(x)* of the horizontal straight beam and the beam deflection in the *X* direction *δ*_1_ are determined as

(17) $$\left\{ \begin{array}{l} P(x_{1})=F,0\leq x\leq L_{{}_{1}}\\ \delta_{1}=\int \frac{P_{1}(x)}{ES}\cdot \frac{\partial P_{1}(x)}{\partial F}dx=\frac{FL_{1}}{ES} \end{array}\right.$$

In the 2–3 section of the elastic beam, the bending moment *M*_2_*(x)* of the circular beam with radius *r* and the beam deflection in the *X* direction *δ*_2_ are expressed as

(18) $$\left\{ \begin{array}{l} M_{2}(x)=Fr(1-\sin\alpha ),0\leq \alpha \leq \pi /2\\ \delta_{2}=\int \frac{M_{2}(x)}{EI_{o}}\cdot \frac{\partial M_{2}(x)}{\partial F}dx=\frac{Fr}{4EI_{o}}(3\pi r-8r) \end{array}\right.$$

In the 3–4 section of the elastic beam, the bending moment *M*_3_*(x)* of the vertical beam and the beam deflection in the *X* direction *δ*_3_ are given by

(19) $$\left\{ \begin{array}{l} M_{3}(x)=Fx,r\leq x\leq r+L\\ \delta_{3}=\int \frac{M_{3}(x)}{EI_{o}}\cdot \frac{\partial M_{3}(x)}{\partial F}dx=\frac{F\left[{\left(r+L\right)}^{3}-r^{3}\right]}{3EI_{o}} \end{array}\right.$$

In the 4–5 section of the elastic beam, the bending moment *M*_4_*(x)* of the circular beam and the beam deflection in the *X* direction *δ*_4_ are determined as

(20) $$\left\{ \begin{array}{l} M_{4}(x)=F(L+r+R\sin\alpha ),0\leq \alpha \leq \pi \\ \delta_{4}=\int \frac{M_{4}(x)}{EI_{o}}\cdot \frac{\partial M_{4}(x)}{\partial F}dx=\frac{F}{2EI_{o}}\left[2\pi {\left(L+r\right)}^{2}+8R(L+r)+\pi R^{2}\right] \end{array}\right.$$

In the 5–6 section of the elastic beam, the bending moment and the radial displacement are the same as those of the 3–4 section and are expressed as follows:

(21) $$\left\{ \begin{array}{l} M_{5}(x)=Fx,r\leq x\leq r+L\\ \delta_{5}=\int \frac{M_{5}(x)}{EI_{o}}\cdot \frac{\partial M_{5}(x)}{\partial F}dx=\frac{F\left[{\left(r+L\right)}^{3}-r^{3}\right]}{3EI_{o}} \end{array}\right.$$

In the 6–7 section of the elastic beam, the axial tension *P*_6_*(x)* of the straight beam and the beam deflection in the *X* direction *δ*_6_ are obtained as

(22) $$\left\{ \begin{array}{l} P_{6}(x_{1})=F,0\leq x\leq L_{2}\\ \delta_{6}=\int \frac{P_{6}(x)}{ES}\cdot \frac{\partial P_{6}(x)}{\partial F}dx=\frac{FL_{2}}{ES} \end{array}\right.$$

In the working mode vibration, the radial deformation is mainly the bending deformation of the curved beam and the straight beam, and the tensile deformation of the horizontal straight beam is negligible. Therefore, according to linear elasticity theory, the radial deflections of the curved beam and the longitudinal straight beam are superimposed, and the radial stiffness coefficient of the double U-shaped elastic beam is obtained as:

(23) $$\delta_{o}=\sum \delta_{i}$$

According to elastic theory, the radial stiffness coefficient of the double U-shaped elastic beam can be expressed as

(24) $$K_{o}=\frac{F}{\delta_{o}}$$

when the mode number *n* = 2, the stiffness of the DUVRG can be obtained as

(25) $$K_{r}=\frac{E\pi w_{r}^{3}hn^{2}{\left(n^{2}-1\right)}^{2}}{12R(1-\mu^{2})}$$

where *w_r_* is the width of the resonant ring, *R* is the radius of the resonant ring, *h* is the height of the resonant ring and *µ* is the Poisson’s ratio of the silicon material.

The equivalent stiffness of the DUVRG can be expressed as

(26) $$\begin{array}{l} k_{11}=k_{r}+\sum_{i=1,3,5,7}k_{o}\\ k_{22}=k_{r}+\sum_{i=2,4,6,8}k_{o} \end{array}$$

The equivalent masses of the DUVRG in the drive direction and sense direction, respectively, can be obtained as

(27) $$\begin{array}{l} m_{1}=m_{r}+\sum_{i=2,4,6,8}m_{o}+\frac{1}{3}\sum_{i=1,3,5,7}m_{o}\\ m_{2}=m_{r}+\sum_{i=1,3,5,7}m_{o}+\frac{1}{3}\sum_{i=2,4,6,8}m_{o} \end{array}$$

where *m_r_* and *m_o_* are the equivalent masses of the resonant ring and double U-shaped elastic beam, respectively. Finally, the natural frequency of the DUVRG can be given as

(28) $$\omega_{1,2}=\frac{1}{2\pi }\sqrt{\frac{k_{r}+4k_{o}}{m_{r}+4m_{o}+\frac{4}{3}m_{o}}}$$

The material parameters and geometric parameters of the ring resonator in the design and FEA simulation stages are shown in Table 1. These parameters are substituted into the previous equation, and the theoretical operating frequency of the DUVRG is calculated to be 9.6131 kHz.

### 3.2. DUVRG Structure Finite Element Analysis

#### 3.2.1. Mode Simulation

**Figure 6:**
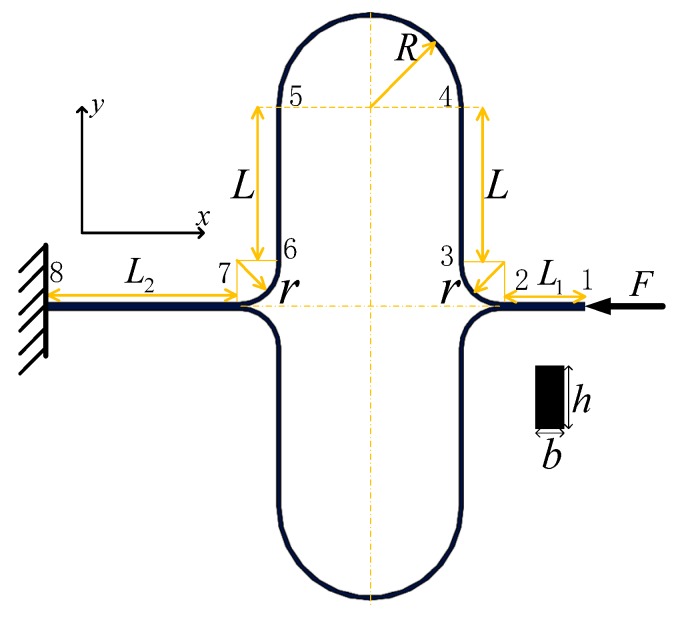
Double-U beam stiffness diagram.

Modal analysis can be used to determine the vibration characteristics of the gyroscope structure, such as the natural frequency, mode shape and vibration stability. After the preliminary design was developed using the theoretical calculation, a more detailed DUVRG geometry was determined by FEA using the commercial software ANSYS-Workbench. The mode shapes and corresponding resonant frequencies for the modes *n* = 1, *n* = 2 and *n* = 3 are shown in Figure 6. The definite design parameters of the DUVRG are shown in Table 1.

**Figure 7:**
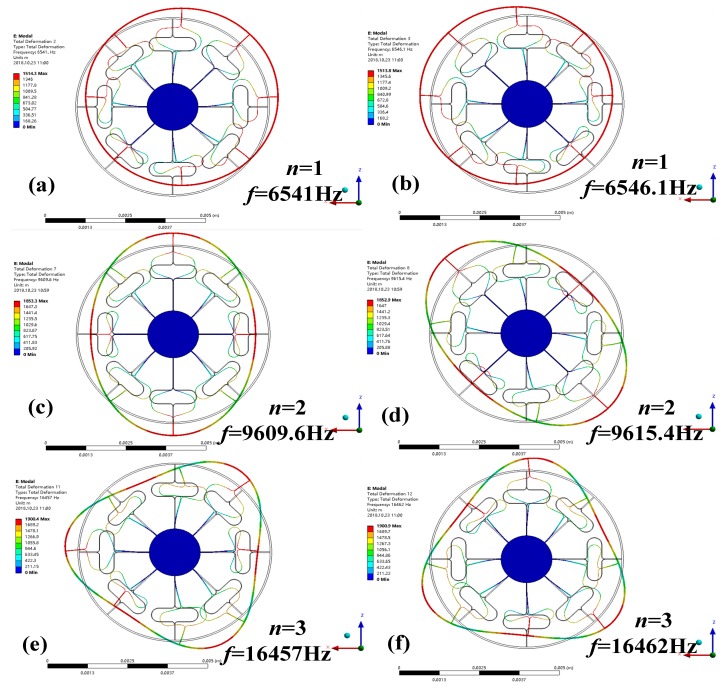
Modal diagram of the mode number *n* = 1,2 and 3.

As shown in Figure 7, modes *n* = 1 are in-plane inflexible motion in the *XOY* plane, and modes *n* = 2 are the “circle-ellipse” in-plane flexural motion in the *XOY* plane; these modes include the drive and sense modes. Modes *n* = 3 are the “circle-triangular circle” in-plane flexural motion in the *XOY* plane, and these modes also include the drive and sense modes. From Figure 7, it can be concluded that the resonant frequencies of the drive and sense modes of the DUVRG are 9.6096 kHz and 9.6154 kHz, respectively. The frequency gap between the drive and sense modes is 5.8 Hz in the finite element simulation. The maximum mode resonant frequency error calculated by the DUVRG stiffness model and FEA is 0.04%.

#### 3.2.2. Harmonic Response Simulation

The main purpose of the harmonic response analysis of the DUVRG is to calculate the displacement response of the resonant structure under the action of the electrostatic force and obtain the amplitude-frequency response curve of the DUVRG. After sweep simulation analysis, the peak frequency of the resonant structure of the DUVRG and the peak value are observed. And the quality factors of drive and sense mode are set at about 28,000 to simulate air damping in the structure.

**Figure 8:**
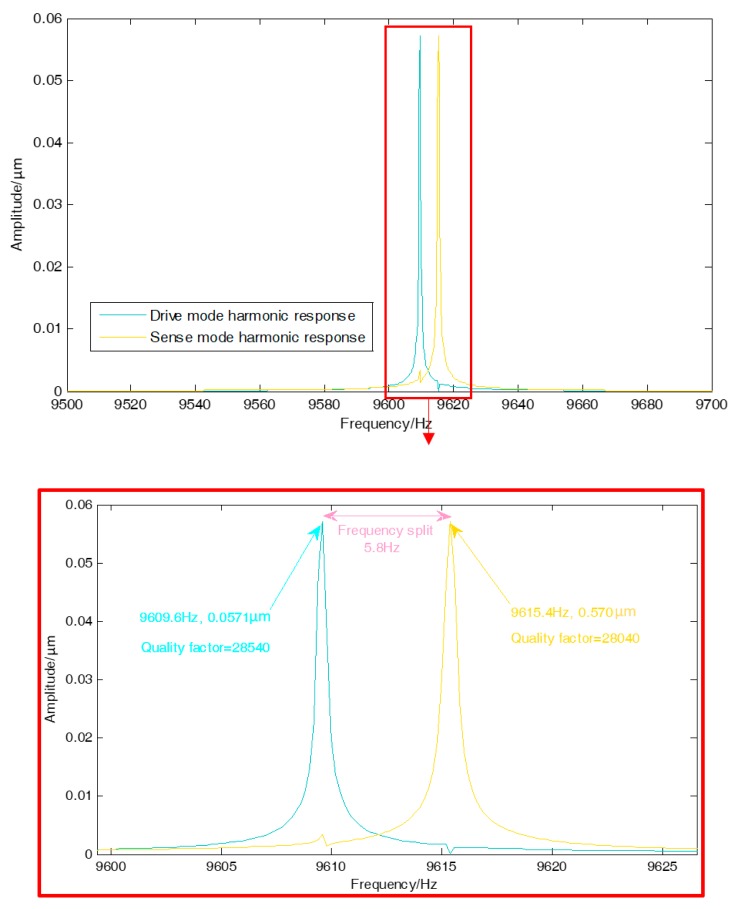
Amplitude frequency response curve of DUVRG.

In the 0° and 180° directions of the ring structure, a simple harmonic force with a relative amplitude of 1 μN is applied. The amplitude-frequency characteristics of the vibration of the ring structure after loading are shown in Figure 8. A response peak point appears at 9609.6 Hz over the entire frequency band, and this frequency is the resonant frequency of the drive mode of the DUVRG. When a harmonic force of 1 μN is applied in the 0° and 180° axis directions, the drive mode in which the resonance frequency is 9609.6 Hz is excited, and the vibration amplitude is about 0.0571 μm.

A simple harmonic force with an amplitude of 1 μN is applied in the 45° axis direction and the 225° axis direction of the ring structure, and the amplitude-frequency characteristics of the ring structure after loading are shown in Figure 8. As shown in the figure, a response peak point occurs at 9615.4 Hz over the entire frequency band, and this frequency is the resonant frequency of the sense mode of the DUVRG. When a harmonic force of 1 μN is applied in the 45° and 135° axis directions, the sense mode in which the resonance frequency is 9615.4 Hz is excited, and the vibration amplitude is 0.0571 μm. The amplitude error between drive and sense modes are supposedly generated by the soft and mesh.

## 4. MEMS DUVRG Structure Fabrication

**Figure 9:**
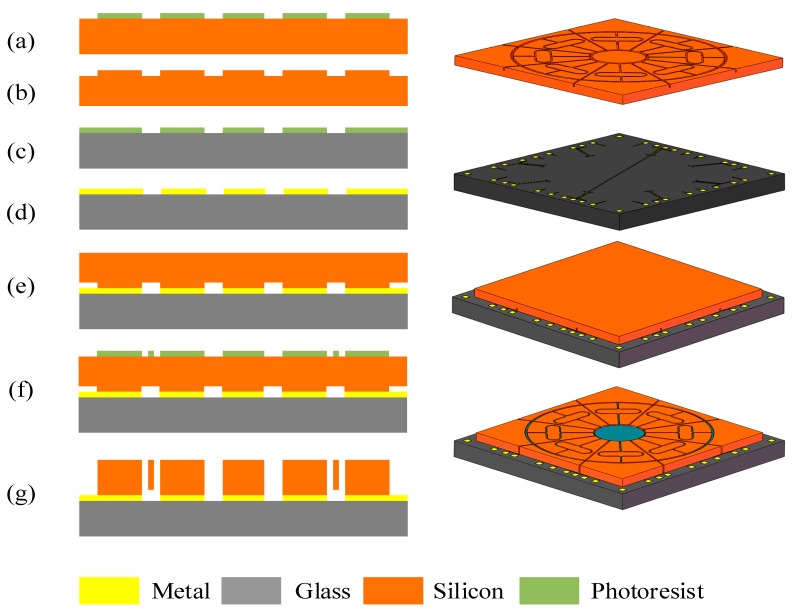
Main process flow of fabrication of DUVRG.

The DUVRG can be fabricated by a conventional silicon-on-glass (SOG) and deep reactive ion etching (DRIE) process. The main device layer is made on a low resistivity, heavily doped 300 µm-thick monocrystalline single crystal silicon wafer. The main process flow is shown in Figure 9.

**Figure 10:**
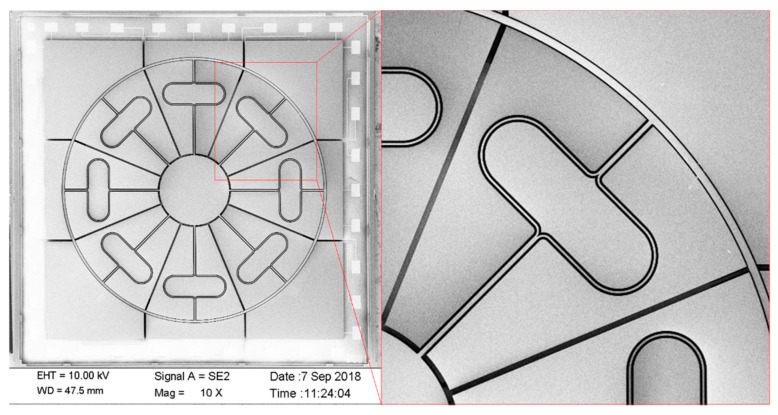
Scanning electron micrograph image of the fabricated DUVRG.

As shown in Figure 9, 300 µm-thick heavily doped monocrystalline silicon wafers are used as silicon structures. A general manufacturing process, including:

(1) sputtering metal and metal patterns on top of the glass layer to form electrode leads on the glass substrate (Figure 9a–c);
(2) etching the bottom surface of the silicon wafer to form supporting anchors and independent electrodes and anodic bonding between the silicon wafer and the glass layer (Figure 9d–f);
(3) etching the silicon wafer by DRIE technology after anodic bonding, so that the resonant structure is released and the capacitor electrode is separated (Figure 9g,h);
(4) etching glass chips to form glass caps to protect the microstructure and anodic bonding of the glass caps with silicon chips, forming a three-layer bonded gyroscopic structure (Figure 9i,k);
(5) the fabrication process succeeded in fabricating a precise structure, and scanning electron microscopy (SEM) images are shown in Figure 10.

## 5. Experiment and Discussion

### 5.1. DUVRG Monitoring System

**Figure 11:**
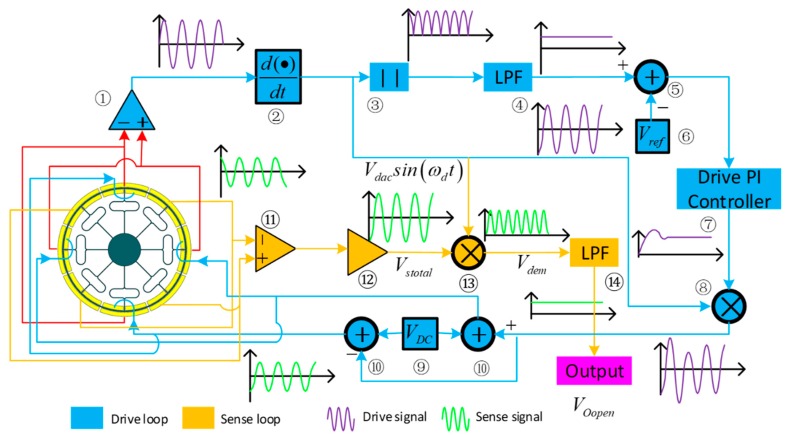
DUVRG system schematic diagram.

The gyroscope control and sense system is shown in Figure 11. In the drive loop, the drive frame displacement *x(t)* is detected by drive sensing combs and picked up by a differential amplifier ①. Then, the signal phase is delayed by 90° (through ②) to satisfy the phase requirement of the AC drive signal *V_dac_*Sin*(ω_d_t)*. Next, *V_dac_*Sin*(ω_d_t)* is processed by a full-wave rectifier ③ and a low pass filter ④. Then, *V_dac_* is compared (in ⑤) with the reference voltage *V_ref_* ⑥. Next, the drive PI controller ⑦ generates the control signal, which is modulated by *V_dac_Sin(ω_d_t)*, and then the signal is superposed (through ⑩) by *V_DC_* ⑨ to the stimulation drive mode.

The sensing system employs an open loop and utilizes the same interface as the drive circuit. First, the sensing signals of the left and right masses are detected separately with a differential sense amplifier ⑪. The output signals are processed by a second differential amplifier ⑫ to generate the signal *V_stotal_*. Then, *V_stotal_* is demodulated by the signal *V_dac_*Sin*(ω_d_t)* (in ⑬). Next, the demodulated signal *V_dem_* passes through the low-pass filter ⑭; thus, the sensing mode’s movement signal *V_Oopen_* can be obtained.

**Figure 12:**
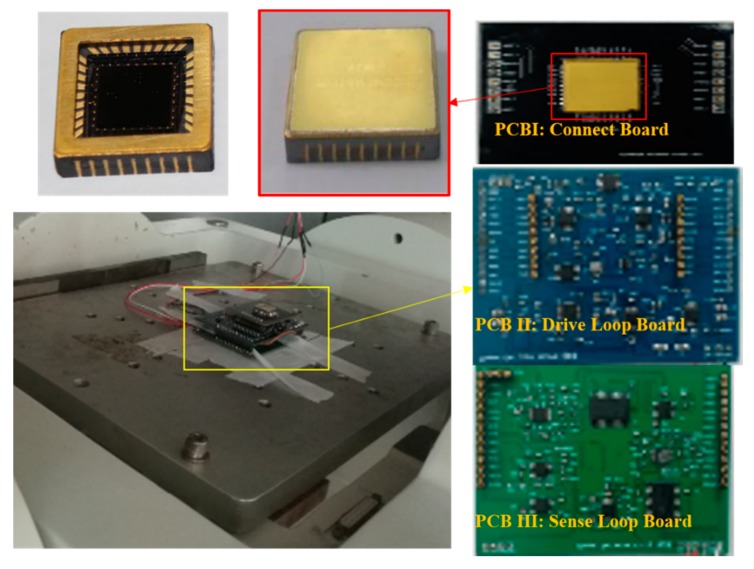
DUVRG prototype.

The DUVRG prototype is shown in Figure 12, and the DUVRG structure is packaged in a vacuum ceramic shell, which is 9.5 mm × 9.5 mm, and the monitoring system is divided into three PCBs: PCBI contains the connecting circuit and connected with structure chip; PCB II is the drive loop, and PCB I inserts on its top face and PCB III inserts on its back face; PCB III contains the sense loop, and the output signal is on PCB III.

### 5.2. DUVRG Test Platform

**Figure 13:**
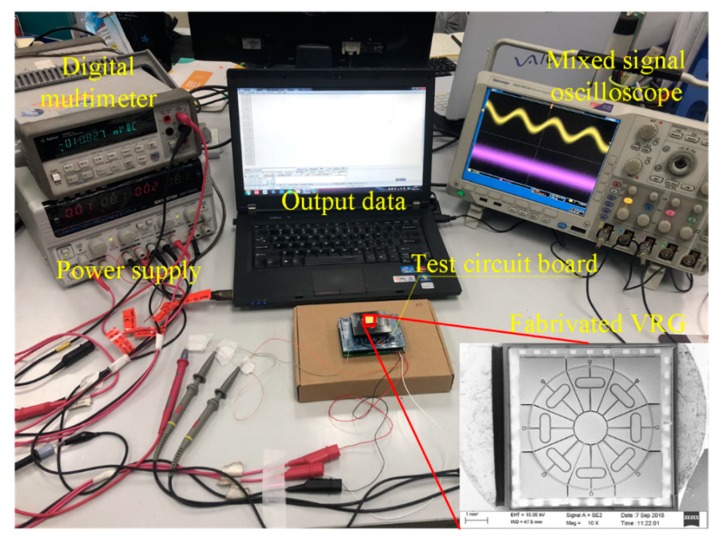
DUVRG bias and frequency test platform.

Prototypes of the DUVRG were tested with atmosphere packaging to evaluate their resonance and performance characteristics. A sample of the completed ring resonance structure (shown in Figure 9) was used to construct a simple working mode frequency response test system in a laboratory environment, as shown in Figure 13. The function signal generator generated an alternating voltage signal containing a bias voltage to be applied to the DUVRG drive electrodes, and then the output voltage signal of the drive feedback electrodes was amplified by an amplifier circuit and then measured by a multimeter (Keysight 34401A, Santa Rosa, CA, USA).

**Figure 14:**
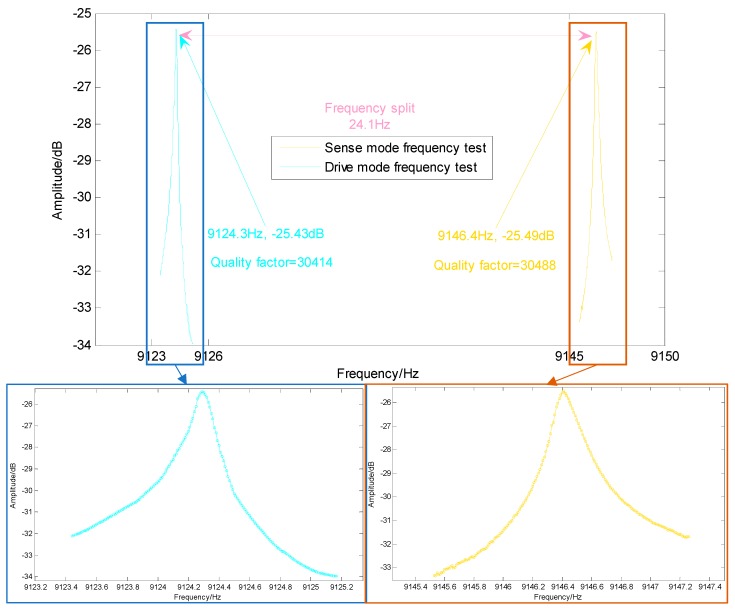
DUVRG structure frequency test results.

The frequency test signal is generated by function signal generator, and the frequency of the applied signal and the voltage amplitude detected by the multimeter are recorded, as shown in Figure 14. The maximum voltage value corresponding to signal frequencies of 9124.3 Hz with the amplitude is −25.43 dB, (drive mode) and 9146.4 Hz with the amplitude is −25.49 dB, (sense mode), which are basically consistent with the theoretical model data, and then the voltage measured by the multimeter began to drop. The measured resonant frequency differed by a maximum of 5.33% from the mathematical analysis and by a maximum of 5.36% from the FEA, and these results verify the accuracy of the theoretical model.

**Figure 15:**
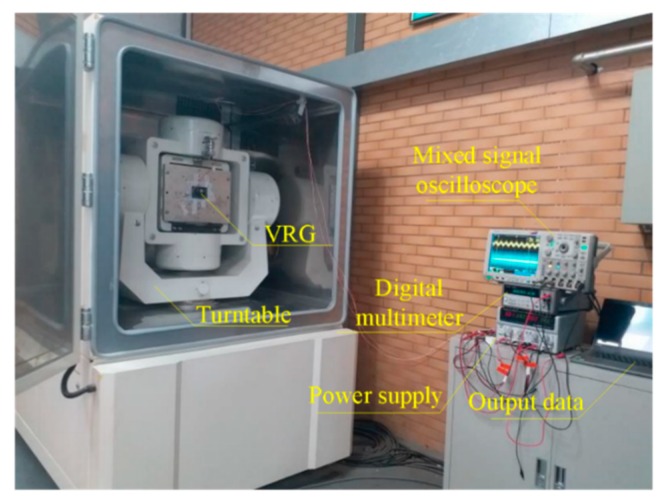
DUVRG scale factor test platform.

**Figure 16:**
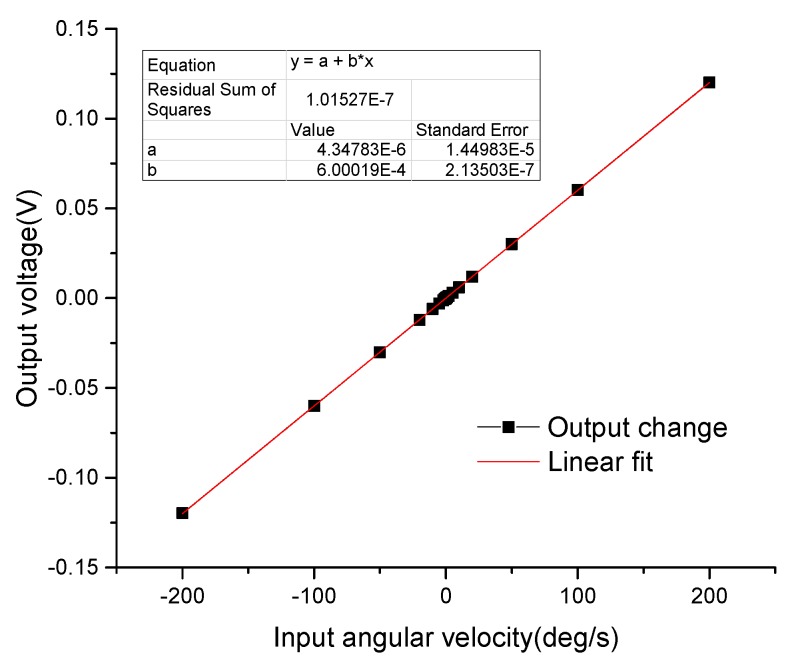
DUVRG scale factor test result.

The DUVRG prototype was fixed on the turntable test system (as shown in Figure 15) for scale factor testing. The rate sensitivity was measured under a rotating disk at input angular rates of 0 (°)/s, ±0.1 (°)/s, ±0.2 (°)/s, ±0.5 (°)/s, ±1 (°)/s, ±2 (°)/s, ±5 (°)/s, ±10 (°)/s, ±20 (°)/s, ±50 (°)/s, ±100 (°)/s, ±150 (°)/s, ±200 (°)/s and the output values at each point were recorded. The results of this test are shown in Figure 16. The measured nonlinearity of the scale factor was found to be negligible, and the linear relation can be approximately expressed by

(29) $$V_{o}=k\Omega +V_{so}=6.00\times 10^{-4}\Omega +4.35\times 10^{-6}$$

**Figure 17:**
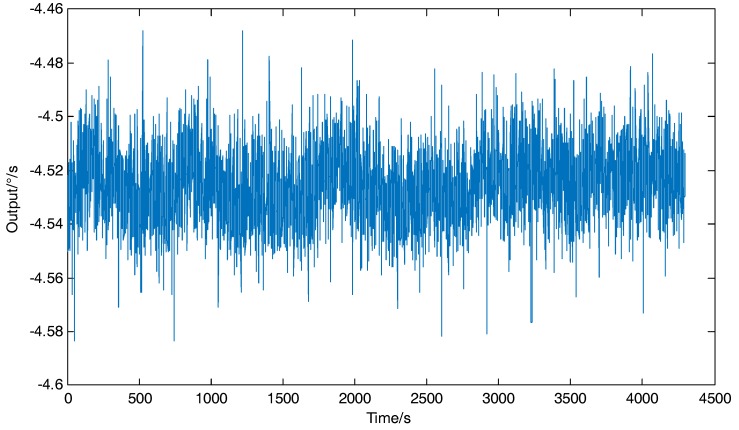
DUVRG static output curve.

**Figure 18:**
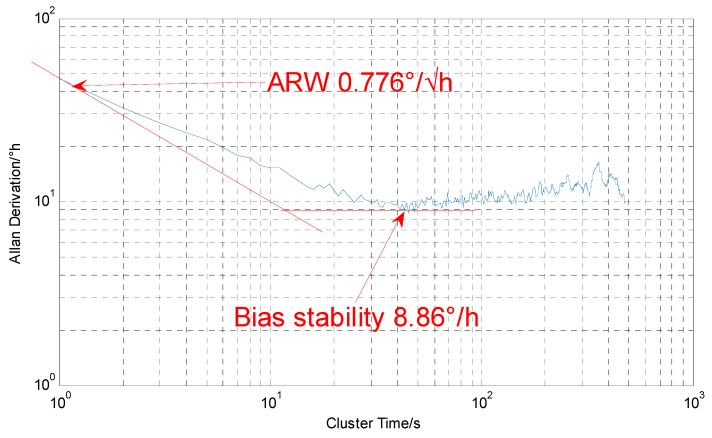
DUVRG Allan derivation result.

The DUVRG was tests with static platform and the sampling rate is 1 Hz, the test process lasts 4800 s and the tested curve is shown in Figure 17. The Allan variance analysis results for the output data of the DUVRG prototype are shown in Figure 18. From the Allan variance curve, we can determine that the bias instability was approximately 8.86 (°)/h and the angular random walk (ARW) was approximately 0.776 (°)/√h.

## 6. Conclusions and Discussions

In this study, a new microelectromechanical system, a vibration ring gyroscope with a double U-beam (DUVRG) was proposed. Both a stiffness model and finite element analyses (FEAs) were used to design the double U-beam for the DUVRG. Then, DUVRG structures were manufactured by an efficient fabrication process using silicon-on-glass (SOG) and deep reactive ion etching (DRIE). The vacuum package was also verified and the quality factors of the gyroscope structure are tested to be more than 30,000. The performance and resonance characteristics of the DUVRG prototype were tested, and the maximum relative frequency errors between the test results and the FEA results and theoretical calculation results were 5.33% and 5.36%, respectively. The Allan variance of the static test data for the DUVRG at room temperature demonstrated that the bias instability was 8.86 (°)/h and the angular random walk (ARW) was 0.776 (°)/√h, and the scale factor was 6.00 mV/(°)/s in the full-scale input range of ±200 (°)/s. These test results verify the high performance of the proposed DUVRG.

Based on the above analysis, the rationality of the design of the ring structure is proved, especially in the gyroscope’s structural consistency, frequency matching, bandwidth, sensitivity and zero-bias stability. These results laid the theoretical foundation for further research and fabrication of high performance MEMS VRG. The anti-shock characteristic of the DUVRG will be investigated and tested in future work, and the anti-shock amplitude value of the structure is sanguine to be more than 20,000 g.

Compared to conventional HRG, the novel structural VRG simplify the fabrication process and improve fabrication precision, making it worthwhile to research and develop further. The accuracy and noise performance of the DUVRG are not ideal. They need to be further improved by zero offset suppression or force balance control, work which will be reported in the future.

## Figures and Tables

**Table 1:** MEMS gyroscope structure mechanical value.

Parameter	Value
Elastic Modulus (*E*)	169 GPa
Poisson’s ratio (*µ*)	0.27
Density (*ρ*)	2328.3 kg/m³
Radius of Resonant ring (*R*_O_)	3000 μm
Height of Resonant ring (*h*)	150 μm
Width of Resonant ring (*w*_r_)	50 μm
Width of U-beam (*b*)	15 μm

## References

[1] Tao K., Yi H.P., Tang L.H., Wu J., Wang P.H., Wang N., Hu L.X., Fu Y.Q., Miao J.M., Chang H.L. (2019). Piezoelectric ZnO thin films for 2DOF MEMS vibrational energy harvesting. Surf. Coat. Technol..

[2] Guo H., Chen Y., Wu D., Zhao R., Tang J., Ma Z., Xue C., Zhang W., Liu J. (2017). Plasmon-enhanced sensitivity of spin-based sensors based on a diamond ensemble of nitrogen vacancy color centers. Opt. Lett..

[3] Wang Z., Wang J., Du W. (2018). Research on Fault Diagnosis of Gearbox with Improved Variational Mode Decomposition. Sensors.

[4] Xia D., Yu C., Kong L. (2014). The development of micromachined gyroscope structure and circuitry technology. Sensors.

[5] Guo X., Sun C., Wang P., Huang L. (2018). Vision sensor and dual MEMS gyroscope integrated system for attitude determination on moving base. Rev. Sci. Instrum..

[6] Xu Y., Tian G., Chen X. (2018). Enhancing INS/UWB integrated position estimation using federated EFIR filtering. IEEE Access.

[7] Gao J., Yan G., Shi Y., Cao H., Huang K., Jun L. (2019). Optimization design of Extensor for Improving Locomotion Efficiency of Inchworm-like Capsule Robot. Sci. China Technol. Sci..

[8] Guo X., Tang J., Li J., Wang C., Shen C., Liu J. (2019). Determine turntable coordinate system considering its non-orthogonality. Rev. Sci. Instrum..

[9] Wang Z., Zhou J., Wang J. (2019). A novel Fault Diagnosis Method of Gearbox Based on Maximum Kurtosis Spectral Entropy Deconvolution. IEEE Access.

[10] Guo H., Zhu Q., Tang J., Nian F., Liu W., Zhao R., Du F., Yang B., Liu J. (2018). A temperature and humidity synchronization detection method based on microwave coupled-resonator. Sens. Actuators B.

[11] Huang H., Chen X., Zhang J. (2017). Weight self-adjustment adams implicit filtering algorithm for attitude estimation applied to underwater gliders. IEEE Access.

[12] Shen Q., Chang H., Wu Y., Xie J. (2019). Turn-on bias behavior prediction for micromachined Coriolis vibratory gyroscopes. Measurement.

[13] Ding X., Zhu K., Li H. (2017). A Switch-bridge Based Readout Circuit for Differential Capacitance Measurement in MEMS Resonators. IEEE Sens. J..

[14] Cao H., Li H., Liu J., Shi Y., Tang J., Shen C. (2016). An improved interface and noise analysis of a turning fork microgyroscope structure. Mech. Syst. Signal Process..

[15] Zotov S., Simon S., Prikhodko I., Trusov A., Shkel A. (2014). Quality Factor Maximization Through Dynamic Balancing of Tuning Fork Resonator. IEEE Sens. J..

[16] Xie J., Chen L., Xie H., Zhou J., Liu G. (2018). The Application of Chemical Foaming Method in the Fabrication of Micro Glass Hemisphere Resonator. Micromachines.

[17] Cao H., Li H., Shao X., Liu Z., Kou Z., Shan Y., Shi Y., Shen C., Liu J. (2018). Sensing mode coupling analysis for dual-mass MEMS gyroscope and bandwidth expansion within wide-temperature range. Mech. Syst. Signal Process..

[18] Cao H., Zhang Y., Han Z., Shao X., Gao J., Huang K., Shi Y., Tang J., Shen C., Liu J. (2019). Pole-Zero-Temperature Compensation Circuit Design and Experiment for Dual-mass MEMS Gyroscope Bandwidth Expansion. IEEE/ASME Trans. Mechatron..

[19] Shen C., Song R., Li J., Zhang X., Tang J., Shi Y., Liu J., Cao H. (2016). Temperature drift modeling of MEMS gyroscope based on genetic-Elman neural network. Mech. Syst. Signal Process..

[20] Cao H., Zhang Y., Shen C., Liu Y., Wang X. (2018). Temperature Energy Influence Compensation for MEMS Vibration Gyroscope Based on RBF NN-GA-KF Method. Shock Vib..

[21] Shen C., Yang J., Tang J., Liu J., Cao H. (2018). Note: Parallel processing algorithm of temperature and noise error for micro-electromechanical system gyroscope based on variational mode decomposition and augmented nonlinear differentiator. Rev. Sci. Instrum..

[22] Cao H., Li H., Kou Z., Shi Y., Tang J., Ma Z., Shen C., Liu J. (2016). Optimization and Experimentation of Dual-Mass MEMS Gyroscope Quadrature Error Correction Methods. Sensors.

[23] Li Q., Xiao D., Zhou X., Xu Y., Zhuo M., Hou Z., He K., Zhang Y., Wu X. (2018). 0.04 degree-per-hour MEMS disk resonator gyroscope with high-quality factor (510 k) and long decaying time constant (74.9 s). Microsyst. Nanoeng..

[24] Mamo Y. (2005). Fabrication of High Aspect Ratio Vibrating Cylinder Microgyroscope Structures by Use of the LIGA Process. Ph.D. Thesis.

[25] Xi X., Wu X., Wu Y., Zhang Y., Tao Y., Zheng Y., Xiao D. (2013). Structural-Acoustic Coupling Effects on the Non-Vacuum Packaging Vibratory Cylinder Gyroscope. Sensors.

[26] Casinovi G., Norouzpour-Shirazi A., Dalal M., Farrokh A. (2016). Gyroscope sensing and self-calibration architecture based on signal phase shift. Sens. Actuators A Phys..

[27] Su Z., Fu M., Li Q., Liu N., Liu H. (2013). Research on Bell-Shaped Vibratory Angular Rate Gyro’s Character of Resonator. Sensors.

[28] Wang R., Bai B., Feng H., Ren Z., Cao H., Xue C., Zhang B., Liu J. (2016). Design and Fabrication of Micro Hemispheric Shell Resonator with Annular Electrodes. Sensors.

[29] Ayazi F., Najafi K. Design and fabrication of high-performance polysilicon vibrating ring gyroscope. Proceedings of the 11th International Workshop on MICRO Electro Mechanical Systems.

[30] Ayzai F., Najafi K. (2000). High aspect-ratio polysilicon micromachining technology. Sens. Actuators A.

[31] Yoon S., Park U., Rhim J., Yang S.S. (2015). Tactical grade MEMS vibrating ring gyroscope with high shock reliability. J. Microelectron. Eng..

[32] Liu J.L., Chen D.Y., Wang J.B. (2014). Fabrication and test of an electromagnetic vibrating ring gyroscope based on SOI wafer. J. Electron..

[33] Zaman M.F., Sharma A., Ayazi F. (2009). The Resonating Star Gyroscope: A Novel Multiple-Shell Silicon Gyroscope with Sub-5 deg/hr Allan Deviation Bias Instability. IEEE Sens. J..

[34] Hu Z.X., Gallacher B.J., Burdess J.S., Fell C.P., Townsend K. (2011). A parametrically amplified MEMS rate gyroscope. Sens. Actuators A Phys..

[35] Tao Y., Wu X., Xiao D., Wu Y., Cui H., Xi X., Zhu B. (2011). Design, analysis and experiment of a novel ring vibratory gyroscope. Sens. Actuators A.

[36] Zhou X., Wu Y., Wu X., Zhang Y., Zheng Y. (2016). A novel ring vibrating gyroscope based on side piezo-electrodes. J. Cent. South Univ..

[37] Kou Z., Liu J., Cao H., Shi Y., Ren J., Zhang Y. (2017). A novel MEMS S-springs vibrating ring gyroscope with atmosphere package. AIP Adv..

[38] Kou Z., Liu J., Cao H., Han Z., Sun Y., Shi Y., Ren S., Zhang Y. (2018). Investigation, modeling, and experiment of an MEMS S-springs vibrating ring gyroscope. J. Micro/Nanolithogr. MEMS MOEMS.

